# Presenting Characteristics, Comorbidities, and Outcomes Among Patients With COVID-19 Hospitalized in Pakistan: Retrospective Observational Study

**DOI:** 10.2196/32203

**Published:** 2021-12-14

**Authors:** Hashaam Akhtar, Sundas Khalid, Fazal ur Rahman, Muhammad Umar, Sabahat Ali, Maham Afridi, Faheem Hassan, Yousef Saleh Khader, Nasim Akhtar, Muhammad Mujeeb Khan, Aamer Ikram

**Affiliations:** 1 Yusra Institute of Pharmaceutical Sciences Islamabad Pakistan; 2 School of Chemical and Materials Engineering National University of Science and Technology Islamabad Pakistan; 3 Department of Medicine Benazir Bhutto Hospital Rawalpindi Pakistan; 4 Centre for Liver and Digestive Diseases Holy Family Hospital Rawalpindi Medical University Rawalpindi Pakistan; 5 Department of Gynecology and Obstetrics Pakistan Air Force Hospital Islamabad Pakistan; 6 Department of Biotechnology Quaid I Azam University Islamabad Pakistan; 7 CT Angiography Department Armed Forces Institute of Cardiology Rawalpindi Pakistan; 8 Department of Community Medicine, Public Health and Family Medicine Faculty of Medicine Jordan University of Science & Technology Irbid Jordan; 9 Department of Infectious Diseases Pakistan Institute of Medical Sciences Islamabad Pakistan; 10 Department of Infectious Diseases Holy Family Hospital Rawalpindi Medical University Rawalpindi Pakistan; 11 Department of Virology National Institutes of Health Islamabad Pakistan

**Keywords:** COVID-19, indicators, symptoms, risk factors, comorbidities, severity, Pakistan

## Abstract

**Background:**

COVID-19 became a pandemic rapidly after its emergence in December 2019. It belongs to the coronavirus family of viruses, which have struck a few times before in history. Data based on previous research regarding etiology and epidemiology of other viruses from this family helped played a vital role in formulating prevention and precaution strategies during the initial stages of this pandemic. Data related to COVID-19 in Pakistan were not initially documented on a large scale. In addition, due to a weak health care system and low economic conditions, Pakistan’s population, in general, already suffers from many comorbidities, which can severely affect the outcome of patients infected with COVID-19.

**Objective:**

COVID-19 infections are coupled with a manifestation of various notable outcomes that can be documented and characterized clinically. The aim of this study was to examine these clinical manifestations, which can serve as indicators for early detection as well as severity prognosis for COVID-19 infections, especially in high-risk groups.

**Methods:**

A retrospective observational study involving abstraction of demographic features, presenting symptoms, and adverse clinical outcomes for 1812 patients with COVID-19 was conducted. Patients were admitted to the four major hospitals in the Rawalpindi-Islamabad region of Pakistan, and the study was conducted from February to August 2020. Multivariate regression analysis was carried out to identify significant indicators of COVID-19 severity, intensive care unit (ICU) admission, ventilator aid, and mortality. The study not only relates COVID-19 infection with comorbidities, but also examines other related factors, such as age and gender.

**Results:**

This study identified fever (1592/1812, 87.9%), cough (1433/1812, 79.1%), and shortness of breath (998/1812, 55.1%) at the time of hospital admission as the most prevalent symptoms for patients with COVID-19. These symptoms were common but not conclusive of the outcome of infection. Out of 1812 patients, 24.4% (n=443) required ICU admission and 21.5% (n=390) required ventilator aid at some point of disease progression during their stay at the hospital; 25.9% (n=469) of the patients died. Further analysis revealed the relationship of the presented symptoms and comorbidities with the progression of disease severity in these patients. Older adult patients with comorbidities, such as hypertension, diabetes, chronic kidney disease, and asthma, were significantly affected in higher proportions, resulting in requirement of ICU admission and ventilator aid in some cases and, in many cases, even mortality.

**Conclusions:**

Older adult patients with comorbidities, such as hypertension, diabetes, asthma, chronic obstructive pulmonary disorder, and chronic kidney disease, are at increased risk of developing severe COVID-19 infections, with an increased likelihood of adverse clinical outcomes.

## Introduction

COVID-19 is a worldwide pandemic that has proliferated across the globe since its spread from Wuhan, China, in December 2019 [1-3]. Since then, full clinical presentations of this viral infection are still not fully understood, as being an RNA virus with many variants, its clinical manifestations are always different [4]. Nevertheless, cough, fever, dyspnea, body ache, fatigue, and pneumonia are some of the most presented symptoms; however, due to its contagious nature and a wide array of asymptomatic, mild to severe, and, in some cases, life-threatening clinical manifestations [5], as well as a dearth of a standardized effective treatment strategies [6], the COVID-19 case count is escalating with time. According to the World Health Organization (WHO), Pakistan hastily entered the race of having the maximum COVID-19 cases per day and could be classified as having Level 4 COVID-19 transmission, where community transmission of the virus is the main reason for increasing COVID-19 cases [7]. Despite its proximity with China, India, and Iran, the total number of confirmed cases in Pakistan has never risen above 7000 per day, and the death toll per day has also been under control [8].

As of December 11, 2020, the total number of COVID-19 cases worldwide was 71,070,927, with 49,384,495 recoveries; 1,594,772 deaths; and 20,091,660 active cases awaiting outcomes [9]. In Pakistan as of December 11, 2020, the total number of COVID-19 cases was 432,327,149, with 379,092 recoveries; 8653 deaths; and 44,582 active cases awaiting outcomes [8]. The twin cities Rawalpindi and Islamabad in Pakistan have a combined population of around 3 million people and were a hot spot during various waves of COVID-19 [8]. A few small-scale studies have been reported from Pakistan, but there is a dire need to regularly gather, document, and analyze epidemiological data from patients with COVID-19 in Pakistan in a systematic way; this is needed in order to identify high-risk groups and determine risk factors associated with poor disease prognoses, in relation with resultant morbidity or mortality [10-14].

There is a lack of standardized treatment regimens because of varying symptoms associated with COVID-19 infections. The treatment so far has been symptomatic or supportive therapy instead of fixed regimens. A retrospective cohort study on hospitalized patients in China revealed that more men (median age 56 years) than women required intensive care unit (ICU) facilities and had a 28% mortality rate [15]. Nevertheless, health care conditions, prevalence of comorbidities, and lifestyle in Pakistan are quite different than in other countries, in general. We hereby performed a retrospective analysis study aimed at describing the demographic and clinical characteristics and subsequent clinical outcomes that are associated with COVID-19 infections; our cohort included 1812 cases confirmed by real-time reverse transcription–polymerase chain reaction (rRT-PCR) and that were admitted to the four major hospitals in the Rawalpindi-Islamabad region of Pakistan from February to August 2020. The objective was to identify the clinical outcomes and determine the impact of various factors, such as age, gender, and number and types of underlying comorbidities in patients with COVID-19, that can resultantly contribute to adverse clinical outcomes, including COVID-19 severity, requirement of ICU admission, ventilator aid, and mortality.

We analyzed the characteristics and outcomes of patients; these were correlated with the number and types of comorbidities as indicators of COVID-19 severity and prognostic values in pandemic viral infectious diseases, such as COVID-19. More studies are required to assess the clinical manifestations associated with COVID-19 infections, as well as the time and duration of each symptom after viral invasion, in order to provide concrete data about the course of action needed to counter or avoid symptoms by taking necessary prophylactic steps, especially among high-risk patients.

## Methods

This retrospective study was conducted using clinical data acquired from 1812 patients with confirmed COVID-19 who were admitted to four major tertiary care hospitals in Islamabad-Rawalpindi from February to August 2020. The Islamabad-based hospitals were Pakistan Air Force Hospital and Pakistan Institute of Medical Sciences Hospital; the Rawalpindi-based hospitals were Holy Family Hospital and Benazir Bhutto Shaheed Hospital. The study was approved by the ethics review board of Rawalpindi Medical University before data collection, and the data were collected with approval from the National Institute of Health, Pakistan, by HA and SA. In addition to this, the data were systematically organized and recorded using a standardized data collection form specifically designed for the study.

Diagnosis of COVID-19 was confirmed using a polymerase chain reaction (PCR) test on nasal and oropharyngeal swab samples taken at the time of admission to the hospital. A detailed medical history was collected for each patient, including age, gender, exposure history, and clinical manifestations of COVID-19, including fever, cough, and respiratory symptoms. A total of 12 comorbidities were each marked as absent or present and these were categorized into four groups: (1) absence of comorbidities, (2) presence of one comorbidity, (3) presence of two comorbidities, and (4) presence of more than two comorbidities. Communicable comorbidities included hepatitis C (Hep C) and tuberculosis (TB). Noncommunicable disease comorbidities included hypertension (HT), diabetes mellitus (DM), cardiovascular diseases (CVDs), asthma, chronic kidney disease (CKD), nervous system disorders (NSDs), chronic obstructive pulmonary disorder (COPD), cancer, allergies, and anemia. A number of other chronic and acute conditions, such as rheumatoid arthritis, typhoid, stomach ulcers, hypothyroidism, and musculoskeletal injuries, were also reported and were grouped together as a 13th category termed “others.”

The study was performed in line with the Declaration of Helsinki. Patients with COVID-19 who had immunological diseases or missing data were excluded from the analysis to avoid any confounding factors affecting the inflammatory markers assessed in this study.

COVID-19 severity was classified into two groups based on symptoms of patients within the first week of COVID-19 infection: (1) mild to moderate, including patients with fever, cough, and oxygen saturation of 90% or greater on room air along with other symptoms consistent with COVID-19, and (2) severe to critical, including patients with dyspnea (ie, oxygen saturation of less than 90% on room air), pneumonia, and varying degrees of respiratory distress (ie, respiratory rate >30 breaths/min), along with other symptoms. Clinical outcomes studied were COVID-19 severity, requirement of ICU admission, requirement of ventilator, and mortality. This classification was based on WHO guidelines regarding the clinical management of patients with COVID-19 [5].

Chest radiographs and computed tomography scans, including presence or absence of ground-glass or crazy-paving appearance, were noted. Moreover, hematological and biochemical parameters, including blood complete picture, serum ferritin, liver function test, creatinine, and a few others, were also recorded to assist in categorizing the patients. PCR test results of the patients and clinical outcomes, such as admission to ICU, requirement for noninvasive or invasive ventilation provided at the hospital at some stage of disease progression, and mortality, were also recorded.

Statistical analysis was conducted using SPSS Statistics for Windows (version 24; IBM Corp). Categorical variables were described using frequencies and percentages. We used *t* tests to compare two sets of quantitative data. Chi-square tests and Fisher exact tests were used to compare percentages of qualitative variables, where appropriate. Multivariate logistic regression analyses were carried out to determine the indicators of COVID-19 severity and mortality. *P* values of <.05 were considered statistically significant.

## Results

This retrospective study included a total of 1812 patients; 69.2% (n=1253) of the patients were male. Patients included in the study ranged in age from 1 to 79 years, with a mean age of 47.32 years. The percentage of patients in the severe to critical group increased with age: 9.8% (31/315) were less than 30 years of age, 59.8% (177/296) were 60 to 69 years of age, and 74.5% (155/208) were 70 years of age or older. Nevertheless, of the 1812 patients with COVID-19 who were admitted to the one of the four hospitals, upon admission, 1153 (63.6%) fell into the category of mild to moderate COVID-19 infection, whereas 659 (36.4%) patients fell into the severe to critical category. Of these 659 patients, some went on to require admission to ICU (n=443) or ventilator support (n=390) during their stay at the hospital, some recovered and were discharged, and some passed away (n=469). Interestingly, we observed equivalent prevalence of male and female patients in the mild to moderate and the severe to critical categories of patients with COVID-19.

In addition to this, regarding the frequency of comorbidities among 1812 patients, 884 patients (48.8%) had none, 364 patients (20.1%) had one, 335 patients (18.5%) had two, and 229 patients (12.6%) had more than two. The most prevalent comorbidity was HT (n=625, 34.5%), followed by DM (n=532, 29.4%), CVDs (n=243, 13.4%), asthma (n=93, 5.1%), CKD (n=93, 5.1%), Hep C (n=84, 4.6%), TB (n=35, 1.9%), NSDs (n=30, 1.7%), COPD (n=18, 1.0%), cancer (n=13, 0.7%), allergies (n=13, 0.7%), and anemia (n=12, 0.7%) (Table 1).

Moreover, the prevalence of comorbidity stayed higher than 60% for all types in the severe to critical COVID-19 infection category. The prevalence was significant for all types of comorbidities included in this study, except for allergies. Surprisingly, only 111 patients out of 884 (12.6%) with no comorbidities were admitted to hospital or moved into the severe to critical category; this increased to 48.4% (176/364) for patients with a single comorbidity, 60.9% (204/335) for patients with two comorbidities, and 72.5% (166/229) for patients with more than two comorbidities; these results were significant. Table 1 presents the complete picture of COVID-19 severity in patients with respect to age, gender, and the number and types of comorbidities.

**Table 1:** Basic demographic characteristics and COVID-19 severity in hospitalized patients.

Variables		Total cases (N=1812), n (%)^a^	COVID-19 severity, n (%)^b^			*P* value
			Mild to moderate	Severe to critical		
Cases (N=1812)		1812 (100)	1153 (63.6)	659 (36.4)	N/A^c^	
**Age (years)**						
	<30	315 (17.4)	284 (30.2)	31 (9.8)	<.001^d^	
	30-49	653 (36.0)	510 (78.1)	143 (21.9)		
	50-59	340 (18.8)	187 (55.0)	153 (45.0)		
	60-69	296 (16.3)	119 (40.2)	177 (59.8)		
	≥70	208 (11.5)	53 (25.5)	155 (74.5)		
**Gender**						
	Male	1253 (69.2)	801 (63.9)	452 (36.0)	.70	
	Female	559 (30.8)	352 (63.0)	207 (37.0)		
**Number of comorbidities**						
	None	884 (48.8)	773 (87.4)	111 (12.6)	<.001	
	1	364 (20.1)	186 (51.1)	176 (48.4)		
	2	335 (18.5)	131 (39.1)	204 (60.9)		
	>2	229 (12.6)	63 (27.5)	166 (72.5)		
**Type of comorbidity**						
	Hypertension	625 (34.5)	248 (39.7)	377 (60.3)	<.001	
	Diabetes mellitus	532 (29.4)	201 (37.8)	331 (62.2)	<.001	
	Cardiovascular diseases	243 (13.4)	80 (32.9)	163 (67.1)	<.001	
	Asthma	93 (5.1 )	29 (31.2)	64 (68.8)	<.001	
	Chronic kidney disease	93 (5.1)	32 (34.4)	61 (65.6)	<.001	
	Hepatitis C	84 (4.6)	33 (39.3)	51 (60.7)	<.001	
	Tuberculosis	35 (1.9)	11 (31.4)	24 (68.6)	<.001	
	Nervous system disorders	30 (1.7)	7 (23.3)	23 (76.7)	<.001	
	Chronic obstructive pulmonary disorder	18 (1.0)	2 (11.1)	16 (88.9)	<.001	
	Cancer	13 (0.7)	4 (30.8)	9 (69.2)	.01	
	Allergies	13 (0.7)	5 (38.5)	8 (61.5)	.06	
	Anemia	12 (0.7)	2 (16.7)	10 (83.3)	.001	
	Others	84 (4.6 )	22 (26.2)	62 (73.8)	<.001	

^a^Percentages in this column are based on the total number of patients (N=1812).

^b^Percentages in these columns are based on the number of patients reported in the respective rows in the “Total cases” column.

^c^N/A: not applicable; a *P* value was not calculated for this item.

^d^The *P* value for a group of variables is reported in the top row of that group.

The frequency of clinical manifestations in the form of fever, cough, dyspnea, anosmia, ageusia, sore throat, and others are shown in Table 2; fever was the most common symptom among 1812 patients (n=1592, 87.9%), followed by cough (n=1433, 79.1%) and dyspnea (n=998, 55.1%). Loss of taste and smell (n=809, 44.6%), sore throat (n=499, 27.5%), and body aches (n=490, 27.0%) were comparatively less prevalent symptoms.

Indicators of severity varied among 1812 patients included in this study. A total of 443 (24.4%) patients required ICU admission, and 390 (21.5%) required ventilator aid at some point of disease progression during their stay at the hospital. Nevertheless, 469 (25.9%) patients died with or without ICU admission or ventilator aid.

A significant increase in ICU admissions, ventilator aid events, and mortality was observed with an increase in age and number of comorbidities. No significant differences in ICU admission, ventilator aid events, and mortality were observed between males and females. A significant increase in ICU admissions, ventilator aid events, and mortality was observed with the presence of any type of studied comorbidity, except for allergies.

Patients with no known comorbidities had minimal requirements for ICU admission (47/884, 5.3%), ventilator aid events (34/884, 3.8%), and mortality (50/884, 5.7%); these proportions were approximately 10 times higher with patients who had two or more comorbidities. Delving into each type of comorbidity revealed that approximately half of the patients with any type of comorbidity required ICU admission with or without ventilator aid, and a significant number of them expired.

**Table 2:** Prevalence of reported signs and symptoms in patients with COVID-19 at the time of hospital admission.

Signs and symptoms	Patients experiencing these signs and symptoms (N=1812), n (%)
Chest pain	45 (2.5)
Headache	81 (4.5)
Nausea or vomiting	115 (6.3)
Diarrhea	130 (7.2)
Flu	150 (8.3)
Body aches, fatigue, or malaise	490 (27.0)
Sore throat	499 (27.5)
Anosmia or ageusia	809 (44.6)
Dyspnea	998 (55.1)
Cough	1433 (79.1)
Fever	1592 (87.9)

Table 3 shows the clinical outcomes of the hospitalized patients with COVID-19 with respect to age, gender, and number and types of comorbidities.

Multivariate regression analysis was carried out to identify significant indicators of COVID-19 severity, ICU admission, ventilator aid, and mortality. Factors such as age, gender, and number and types of comorbidities were included, except for anemia, which was excluded due to small sample size.

Our results indicate that old age was a significant indicator of COVID-19 severity, ICU admission, ventilator aid, and mortality. Although our data reported a greater number of male patients, male gender had no significant relationship with COVID-19 severity, ICU admission, ventilator aid, and mortality.

In addition, the results of this study revealed that an increase in the number of comorbidities was a significant predictor of COVID-19 severity, ICU admission, ventilator aid, and mortality. HT, DM, COPD, CKD, and asthma were significant predictors COVID-19 severity, ICU admission, ventilator aid, and mortality. NSDs, on the other hand, were a significant predictor of COVID-19 severity, ICU admission, and mortality, but not ventilator aid. TB was a significant predictor of COVID-19 severity and ICU admission, but not ventilator aid and mortality. In addition, Hep C and cancer were both significant predictors of ventilator aid and mortality, whereas CVDs were only a significant predictor of mortality. Presence of allergies was not a significant predictor of any of the study outcomes.

Table 4 shows the multivariate analysis of factors associated with COVID-19 severity, ICU admission, ventilator aid, and mortality.

**Table 3:** Clinical outcomes of patients with COVID-19.

Variables			Total cases (N=1812), n (%)^a^		ICU^b^ admission, n (%)^c^		*P* value		Ventilator aid, n (%)^c^		*P* value		Mortality, n (%)^c^		*P* value
Cases (N=1812)			1812 (100)		443 (24.4)		N/A^d^		390 (21.5)		N/A		469 (25.9)		N/A
**Age (years)**															
	<30	315 (17.4)		22 (7.0)		<.001		17 (5.4)		<.001		19 (6.0)		<.001	
	30-49	653 (36.0)		84 (12.9)		<.001		75 (11.5)		<.001		88 (13.5)		<.001	
	50-59	340 (18.8)		101 (29.7)		<.001		90 (26.5)		<.001		100 (29.4)		<.001	
	60-69	296 (16.3)		126 (42.6)		<.001		109 (36.8)		<.001		135 (45.6)		<.001	
	≥70	208 (11.5)		110 (52.9)		<.001		99 (47.6)		<.001		127 (61.1)		<.001	
**Gender**															
	Male	1253 (69.2)		297 (23.7)		.27^e^		263 (21.0)		.41		313 (25.0)		.19	
	Female	559 (30.8)		146 (26.1)				127 (22.7)				156 (27.9)			
**Number of comorbidities**															
	None	884 (48.8)		47 (5.3)		<.001		34 (3.8)		<.001		50 (5.7)		<.001	
	1	364 (20.1)		124 (34.4)		<.001		110 (30.2)		<.001		126 (34.6)		<.001	
	2	335 (18.5)		141 (42.1)		<.001		128 (38.2)		<.001		147 (43.9)		<.001	
	>2	229 (12.6)		131 (57.2)		<.001		118 (51.5)		<.001		146 (63.8)		<.001	
**Type of comorbidity**															
	Hypertension	625 (34.5)		274 (43.8)		<.001		245 (39.2)		<.001		294 (47.0)		<.001	
	Diabetes mellitus	532 (29.4)		235 (44.2)		<.001		207 (38.9)		<.001		245 (46.1)		<.001	
	Cardiovascular diseases	243 (13.4)		123 (50.6)		<.001		112 (46.1)		<.001		135 (55.6)		<.001	
	Asthma	93 (5.1 )		51 (54.4)		<.001		45 (48.4)		<.001		53 (57.0)		<.001	
	Chronic kidney disease	93 (5.1)		50 (53.8)		<.001		47 (50.5)		<.001		56 (60.2)		<.001	
	Hepatitis C	84 (4.6)		37 (44.0)		<.001		32 (38.1)		<.001		44 (52.4)		<.001	
	Tuberculosis	35 (1.9)		21 (60.0)		<.001		17 (48.6)		<.001		18 (51.4)		<.001	
	Nervous system disorders	30 (1.7)		19 (63.3)		<.001		16 (53.3)		<.001		21 (70.0)		<.001	
	Chronic obstructive pulmonary disorder	18 (1.0)		15 (83.3)		<.001		16 (88.9)		<.001		16 (88.9)		<.001	
	Cancer	13 (0.7)		7 (53.8)		.01		7 (53.8)		.004		9 (69.2)		<.001	
	Allergies	13 (0.7)		6 (46.2)		.07		6 (46.2)		.03		6 (46.2)		.09	
	Anemia	12 (0.7)		10 (83.3)		<.001		10 (83.3)		<.001		10 (83.3)		<.001	
	Others	84 (4.6 )		51 (60.7)		<.001		48 (67.1)		<.001		54 (64.3)		<.001	

^a^Percentages in this column are based on the total number of patients (N=1812).

^b^ICU: intensive care unit.

^c^Percentages in this column are based on the number of patients reported in the respective rows in the “Total cases” column.

^d^N/A: not applicable; a *P* value was not calculated for this item.

^e^The *P* value for a group of variables is reported in the top row of that group.

**Table 4:** Multivariate analysis of factors associated with COVID-19 severity and clinical outcomes of patients with COVID-19.

Covariates			COVID-19 severity				ICU^a^ admission				Ventilator aid					COVID-19 mortality		
			OR^b^ (95% CI)		*P* value		OR (95% CI)		*P* value		OR (95% CI)		*P* value		OR (95% CI)			*P* value
**Age (years) (vs <30 years)**																		
	30-49	1.9 (1.2-2.9)		.004		1.4 (0.8-2.4)		.19		1.6 (0.9-2.9)		.09		1.8 (1.0-3.1)			.04	
	50-59	3.5 (2.2-5.6)		<.001		2.6 (1.5-4.5)		<.001		2.9 (1.7-5.3)		<.001		3.1 (1.8-5.6)			<.001	
	60-69	5.1 (3.2-8.3)		<.001		3.8 (2.2-6.6)		<.001		3.9 (2.1-7.0)		<.001		5.4 (3.0-9.7)			<.001	
	≥70	11.0 (6.4-18.7)		<.001		5.9 (3.3-10.4)		<.001		6.3 (3.4-11.6)		<.001		10.7 (5.9-19.5)			<.001	
Gender (male vs female)			0.9 (0.7-1.2)		.68		1.1 (0.8-1.4)		.55		1.1 (0.8-1.4)		.71		1.2 (0.9-1.5)			.30
**Number of comorbidities (vs no comorbidities)**																		
	1	4.6 (3.4-6.2)		<.001		6.9 (4.7-10.1)		<.001		8.2 (5.3-12.6)		<.001		6.2 (4.3-9.0)			<.001	
	2	6.8 (4.9-9.5)		<.001		8.8 (5.9-13.1)		<.001		10.8 (7.0-16.7)		<.001		8.2 (5.6-12.0)			<.001	
	>2	10.8 (7.2-16.0)		<.001		14.9 (9.7-23.0)		<.001		17.4 (10.8-27.8)		<.001		16.9 (11.0-26.1)			<.001	
**Type of comorbidities (yes vs no)**																		
	Diabetes mellitus	2.3 (1.7-3.0)		<.001		1.8 (1.4-2.4)		<.001		1.7 (1.3-2.2)		<.001		1.6 (1.2-2.2)			<.001	
	Hypertension	1.9 (1.5-2.5)		<.001		2.0 (1.5-2.7)		<.001		2.0 (1.5-2.7)		<.001		2.0 (1.5-2.7)			<.001	
	Cardiovascular diseases	1.4 (1.0-2.0)		.08		1.4 (1.0-1.9)		.08		1.4 (1.0-2.0)		.054		1.5 (1.1-2.2)			.02	
	Chronic kidney disease	2.1 (1.3-3.5)		.005		2.5 (1.5-4.1)		<.001		2.7 (1.6-4.4)		<.001		3.3 (2.0-5.4)			<.001	
	Asthma	2.4 (1.4-4.0)		.001		2.4 (1.5-4.0)		<.001		2.1 (1.3-3.5)		.003		2.4 (1.5-4.0)			<.001	
	Tuberculosis	2.6 (1.1-6.2)		.03		3.3 (1.4-7.5)		.005		2.1 (0.9-4.6)		.08		1.6 (0.7-3.8)			.27	
	Cancer	3.0 (0.7-12.8)		.13		2.7 (0.7-10.1)		.15		3.3 (0.9-12.4)		<.001		6.3 (1.5-26.2)			.01	
	Nervous system disorders	6.7 (2.6-17.6)		<.001		6.2 (2.7-14.7)		<.001		4.5 (2.0-10.4)		.82		8.5 (3.4-21.1)			<.001	
	Allergies	1.0 (0.2-4.3)		.96		0.9 (0.2-3.4)		.84		1.2 (0.3-4.5)		.54		0.7 (0.2-2.7)			.58	
	Hepatitis C	1.4 (0.8-2.4)		.18		1.3 (0.8-2.1)		.39		1.2 (0.7-2.0)		<.001		1.8 (1.1-3.0)			.03	
	Chronic kidney disease	8.2 (1.7-40.3)		.01		10.2 (2.6-40.3)		.001		21.7 (4.5-104.8)		<.001		17.5 (3.6-86.2)			<.001	
	Others	2.8 (1.6-5.1)		<.001		2.9 (1.7-4.9)		<.001		3.0 (1.8-5.1)		<.001		3.2 (1.9-5.5)			<.001	

^a^ICU: intensive care unit.

^b^OR: odds ratio.

## Discussion

### Principal Findings

COVID-19 was declared a pandemic at the start of 2020 after emerging in December 2019 in Wuhan, China [3]. All the information related to COVID-19, including its risk factors, severity, mortality, clinical manifestations, and other complications, was not very clear, especially in Pakistan. We aimed to highlight and discuss some dependent and independent factors related to COVID-19 in this study.

In our study, the most prevalent presenting symptoms at the time of hospital admission were fever, cough, and shortness of breath. Anosmia, ageusia, sore throat, body aches, fatigue, and malaise were relatively less common presenting symptoms, whereas diarrhea, nausea, and vomiting were the least prevalent presenting symptoms reported by patients at the time of admission. One or more comorbidities, especially HT and DM, were more prevalent among older adults according to our results. Mild to moderate COVID-19 symptoms were highly prevalent in younger groups. These findings were in accordance with those of other studies around the world [15-18]. The most prevalent comorbidities were HT, DM, and CVDs, followed by asthma, CKD, Hep C, TB, NSDs, COPD, cancer, allergies, and anemia, among others.

Two of the most significant findings of this study were related to the age and comorbidity of the patients, which can be explained as age-dependent weakened functionality of cell-mediated immunity with a decline in humoral immune support [15]. On the other hand, SARS-CoV-2 enters the host cell via attachment of its structural spike protein to the membrane-bound angiotensin-converting enzyme 2 (ACE-2) receptor of the host [19]. This attachment results in ACE-2 degradation and affects its role in the renin-angiotensin-aldosterone system (RAAS), resulting in abnormalities in maintaining blood pressure and homeostasis of electrolytes in the human body [20]. ACE-2 also catalyzes the conversion of angiotensin II into angiotensin (1-7), which regulates RAAS in the vasoconstriction of blood vessels, increase of sodium reabsorption in kidneys, and stimulation of the hypothalamus, adrenal cortex, and sympathetic nervous system, to activate thirst centers in the brain and increase secretion of antidiuretic hormone, renin, and aldosterone, respectively [21,22]. Pathophysiological changes in RAAS due to distressed ACE-2 levels in patients with comorbid COVID-19 tend to show undesired outcomes, such as high levels of angiotensin II, which leads to angiogenesis, vascular aging, atherosclerosis, inflammation, and fibrosis, leading to diseases like hypertension, renal failure, and cardiac fibrosis [20]. In addition, angiotensin II also interferes with the anti-inflammatory action of insulin, which highlights its deleterious effect in patients with diabetes [23].

Moreover, following cell entry of SARS-CoV-2, a cascade of events leading to replication of viral nucleic acid and release of mature virus particles from the cell ensues, resulting in stimulation of the host’s humoral and cellular immunity. Uncontrolled, systemic release of proinflammatory cytokines and chemokines as a part of the host immune response to SARS-CoV-2 infection results in a highly toxic “cytokine storm,” or cytokine release syndrome, which is another hallmark of COVID-19 [19]. As a result, severe forms of COVID-19 can be described by three main phases: early infection, involvement of lungs, and systemic inflammation [24]. It can be concluded that factors that contribute to downregulation of ACE-2, dysregulation of RAAS, impairment of B- and T-cell immunity, and development of cytokine release syndrome also contribute to COVID-19 severity, ICU admission, ventilator aid, and mortality [22,24-27].

Similarly, several other meta-analyses reported that HT, DM, CVDs, and CKD were independent risk factors in patients with COVID-19 and indicators of poor prognosis [4,17,28,29]. A few small-scale studies from Pakistan investigating COVID-19 severity and mortality also reported similar trends [10-14]. Moreover, our findings are also in line with a few initial epidemiological reports from Wuhan, China, as well as more recent studies investigating the impact of comorbidities on patients with COVID-19 from China, the United States, the United Kingdom, Egypt, Spain, and Italy [17,18,30-36].

Multivariate analysis of results revealed that increased age of patients and the number of comorbidities were significantly associated with increased odds of COVID-19 severity, ICU admission, ventilator aid, and mortality. This establishes the fact that older patients with underlying conditions are not only at a higher risk of developing infection, but may also be at risk of severe progression that requires ventilator aid, which may result in death. No significant differences were found between males and females with respect to the odds of COVID-19 severity, ICU admission, ventilator aid, and mortality. Although HT and DM were the most prevalent among all comorbidities studied, with proportions of 34.5% and 29.4%, respectively, the mortality rates for all comorbidities were significantly higher, and were higher still among patients with two or more comorbidities, with an odds ratio (OR) of 16.9. Nevertheless, all comorbidities examined in this study had relatively higher ORs with respect to ICU admission, ventilator aid, and mortality, emphasizing the importance of considering these factors during COVID-19 treatment protocols.

In short, the COVID-19–associated downregulation of ACE-2, leading to an imbalance of RAAS, combined with an age-dependent impaired immune response and chronic inflammation in older patients, may lead to adverse clinical outcomes. Moreover, the likelihood of the presence of one or multiple chronic underlying comorbidities also increases with age, which may further contribute to a poor prognosis for older patients with COVID-19. Our findings are in line with several studies that have established old age to be a significant predictor of COVID-19 severity, ICU admission, ventilator aid, and mortality [15,37].

### Conclusions

The ever-evolving nature of viruses makes them a difficult target for drug design and targeted therapies. In such confusing times, large studies monitoring several factors that might be associated in disease progression and severity are important for clarifying the picture and helping in the development of effective and targeted drugs and vaccines. Moreover, such studies help in the identification of high-risk groups that might have a more severe disease progression and may need a different treatment regime than others. In the current situation, most of the major government directives and guidelines are designed to help curtail the number of COVID-19 cases. These measures are focused on the following: (1) preventive strategies to control the rate of infection, where people are encouraged to take suitable preventative measures, such as improvement of hand hygiene, use of facial masks, and social distancing, and (2) management strategies, where patients who test positive with COVID-19 and report mild to moderate symptoms are directed to follow isolation protocols at home, are directed to monitor their progress by themselves while staying in contact with health care workers remotely via telephone, and are encouraged to report to hospitals only if severe symptoms manifest [38,39]. Studies that help in the identification of indicators for COVID-19 severity and mortality, specific to the Pakistani population, can help in the development of more refined public awareness programs and preventive strategies to encourage people who belong to high-risk groups to take more stringent preventive measures. Moreover, more sophisticated management strategies can be developed to evaluate risk assessment and ensure that high-risk groups can be identified in time and receive appropriate medical care when required, thereby helping to reduce the overall burden of disease on our already-weak health care infrastructure [40].

## Abbreviations

ACE-2: angiotensin-converting enzyme 2
CKD: chronic kidney disease
COPD: chronic obstructive pulmonary disorder
CVD: cardiovascular disease
DM: diabetes mellitus
Hep C: hepatitis C
HT: hypertension
ICU: intensive care unit
NSD: nervous system disorder
OR: odds ratio
PCR: polymerase chain reaction
RAAS: renin-angiotensin-aldosterone system
rRT-PCR: real-time reverse transcription–polymerase chain reaction
TB: tuberculosis
WHO: World Health Organization
